# Remarks on Gonorrhea

**Published:** 1904-11

**Authors:** W. L. Champion

**Affiliations:** Atlanta, Ga.


### REMARKS ON GONORRHEA.*

BY W. L. CHAMPION, M.D.,

ATLANTA, GA.

While gonorrhea is a local disease, and remains so in a great majority of cases, the gonococccus has been found in various glands and tissues of the body. It is taken up by the blood-vessels and lymphatics and carried to the serous membranes in various localities. I have seen the incision of a urethral structure followed by a severe gonorrheal rheumatism. It happens that meddlesome instrumentation in the acute stage of gonorrhea will produce acute prostatitis or epididymitis. I have now under my care a patient with gonorrhea who developed appendicitis three weeks ago, that in all probability the gonococcus could be found in the appendix if it was removed. The gonococcus has been found in the joints, tendons, muscles, ureters, kidneys, endocardium, pericardium, plura and meninges of the brain and cord. While I never have demonstrated by microscopic examination the involvement of the

*Read before Atlanta Society of Medicine, September 15, 1904.

kidneys, I have had under my observation several cases of acute nephritis that developed during an attack of gonorrhea that unquestionably was an extension of the disease.

Three of these cases have been followed up and will show albumen in the urine at this time. Years ago I was skeptical about gonorrheal rheumatism, but the longer I continue in genito-urinary work the percentage of cases increase. When rheumatism is a complication of gonorrhea it will usually develop with each succeeding attack, and the disease as a rule is not as severe as imflammatory rheumatism from other courses. While it is stated that chronic enlargement of the prostate is not traceable to gonorrhea, it may be a fact that if gonorrhea had never existed there would be fewer prostates to remove. The attention that is now being directed towards the prostate on account of its so frequent involvment during an attack of gonorrhea will in the near future throw more light on this question. If gonorrheal patients are closely questioned it is very noticeable how frequently we find fissures, ulcers and irritable conditions of the rectum associated with the disease. These conditions may not be due directly to an extension of the disease, but produced by the constant irritation and spasm of the deep urethra affecting structures so closely connected to it. The involvement of adjacent structures of the male urethra such as Cowper's glands, prostate and testicles cause very pronounced constitutional symptoms, and although the condition is local the general effect upon the nervous system is frequently of long duration. In highly nervous individuals there is not a disease that makes such an impression on the mind, makes the individual so thoroughly dejected, as a chronic gonorrhea. We see such patients brood over their stubborn, but curable condition, until they unfit themselves for any earthly business and become eyesores to their doctors. But when we reflect upon the little importance the average young man attaches to gonorrhea, and frequently the too hasty discharge of patients as cured by physicians, it is occasionally refreshing to come in contact with such a patient. The effects of gonorrhea are more serious and permanent in the female, and when it has invaded the deep glandular structures, the tubes, ovaries and peritoneal cavity, it is a question whether it is ever cured. The number of cases of gonorrheal ophthalmia, the large number of inmates of the blind asylums, the number of abortions and sterile marriages due to the disease, should alarm the laity and the profession more than an occasional epidemic of smallpox or yellow fever.

A social problem confronts the human race that is as dormant as far as educational, administrative and sanitary control is concerned as tuberculosis was fifty years ago.

The venereal plague that is sapping the mental, moral and physical vitality of the young men of to-day, making invalids of innocent womanhood and armies of blind and syphilitic infants, should attract the attention of the enlightened profession to such an extent that it would inform the public not only of the dangers, but constant daily venereal contagion, more serious in its consequences than any contagious disease reported to health authorities, and a blight upon future generations.

The fear of non-venereal contagious diseases that has enlightened society and benefited public health by administrative and sanitary measures demonstrates the importance—the necessity—of enlightenment in regard to venereal diseases if good is accomplished in lessening the evil. This generation could not throw its energy in a direction already taken to suppress as far as possible a class of diseases more ruinous to the human race than tuberculosis or cancer for which future generations will be more thankful than to institute measures that will lessen gonorrhea and syphilis, the serious importance of which is dawning upon the medical profession.

When we recall measures that have been tried without apparent results, the drawbacks and probable injurious results from those we might suggest or institute, there looms up before us the grave necessity of concerted action carefully prepared and studied measures, that may be slow in action, but lasting in benefits. There could not be suggested an administrative or sanitary measure that would not be criticized and opposed, and its evil effects enlarged upon; after careful study it is yet a question whether either measuse would accomplish good. Still I think it is an open question and I am inclined to believe it will not be in the distant future before administrative measures will be more general in the control and prevention of venereal diseases. As far as suffering and injury to the human race is concerned gonorrhea is five to one more serious than syphilis. If the public could only be enlightened on this question part of the battle would be won. Administrative and sanitary measures have enlightened the public in regard to smallpox, diphtheria, scarlet fever and other contagious diseases. These measures have been an education that could not have been had in any other way. The public can be educated in regard to the serious nature of venereal diseases and the importance of their limitation and control by proper laws instituted that will attract attention to it. "The education of the public in this respect is chiefly in the hands of the medical profession," and we have not been doing our duty in this respect. It does not dawn upon the average young graduate from a medical college the untold train of evils that follow in the wake of a gonorrhea until years after he has received his diploma. This evil could be corrected by having and attaching more importance to the chair of genito-urinary and venereal diseases than is the case at present. Any physician who will treat a gonorrhea without impressing upon the patient the serious nature of the disease and its far-reaching evil consequences is amiss in his duty. Let the public be so well informed that every young man that has had a gonorrhea, whether months or years before, will consult his physician if he contemplates marriage, to ascertain if he is in a condition to take such a step. The patient should always he impressed that so long as "clap shreds" or pus can be found in the urine that the urethra is still diseased and in many cases infection is possible.

It too frequently happens that men with a slight discharge from the urethra tell us they consider the discharge harmless, not knowing the slight warning drop is teeming with thousands of gonococci.

Many physicians of to-day write prescriptions and allow patients to treat themselves, never look at the urine in a glass, in fact never look at the patient's genitals, never make a microscopic examination of the secretions; just ask if there has been any discharge within the past few days, and if not, dismisses the patient as cured. "While the men who marry with uncured gonorrhea are of course directly responsible for the infection of their wives, the medical profession has not appreciated its share of the responsibility. The physician, when consulted, is more apt to err on the side of leniency than on the side of severity in rendering a decision. Either from ignorance, from a careless examination, from a cheerful optimism, and a disposition to be too indulgent to the inclinations of their patients, they are too prone to stretch every point in their favor. They impose the minimum delay, and, with a fallacy of interpretation of every favorable symptom and a disregard of every indication of a contrary nature, they have too often sanctioned a premature consummation ot marriage which is entirely unwarranted by the condition of the patient, that is followed by the most serious consequences to the wife and mother."* There is a rung missing in our professional ladder in regard to this disease, and it should be placed in position.

### SHE DESERVED THE PENSION.

Among the cases in the United States Pension office, many of which has been pushed to a successful issue on less plausible testimony, is a claim for a widow's pension, in which the claimant declares that at one of the battles at the late war (1861-1865) she was a spectator. She happened to stand behind some soldiers, and was wounded in the abdomen by a ball that had previously wounded a soldier in the testicles, from which she became pregnant and was delivered of a boy. What purports to be corroboratory testimony bearing on the case is the allegation that the boy subsequently underwent lithotomy, when a conoidal musket ball was removed from the bladder.—*Medical Jurisprudence, Witthouse & Becker.*

PROFESSOR CZERNY has given a sum of $2,500 to the University of Heidelberg for the foundation of a prize in memory of his father-in-law, the distinguished physician, Adolf Kussmaul. The income of the money will be awarded every third year on Kussmaul's birthday, February 22, for the best therapeutic work done during the previous three years and published in a German book or periodical.

* Social Diseases and Marriages.—Prince A. Morrow, M.D.

